# Melanoacanthoma of the nipple: two cases with unique clinical presentations^؆^

**DOI:** 10.1016/j.abd.2025.501231

**Published:** 2025-11-01

**Authors:** Aysun Sikar Akturk, Esin Diremsizoglu, Nilgün Sayman, Rebiay Kiran, Cigdem Vural

**Affiliations:** a Department of Dermatology, Faculty of Medicine, Kocaeli University, Kocaeli, Turkey; b Department of Pathology, Faculty of Medicine, Kocaeli University, Kocaeli, Turkey

*Dear Editor*

Melanoacanthoma is a rare pigmented variant of seborrheic keratosis that mimics melanoma, posing a diagnostic challenge.^1^ While commonly found on sun-exposed areas, its occurrence in the nipple-areola complex is rarely reported.^2^, ^3^ We present two cases in young female patients with prior UV exposure, emphasizing the need for clinical awareness and the role of dermoscopy and biopsy in diagnosing pigmented lesions in uncommon sites.

A 27-year-old female presented with a progressively enlarging, darkly pigmented lesion on the left nipple, first noted a year ago. She had a history of full-body tanning bed exposure two years prior. Examination revealed two irregularly bordered hyperpigmented patches (1.5 cm and 1 cm) on the nipple-areola complex (Fig. 1A). Dermoscopy showed a pigmented background with circular structures (Fig. 1B). Histopathology confirmed melanoacanthoma with basal melanin hyperpigmentation, epidermal acanthosis, and elongation of rete ridges (Fig. 2A). Superficial cryotherapy was applied in four sessions over three months, resulting in partial regression, with no progression observed at six months of follow-up (Fig. 2B).

**Fig. 1:**
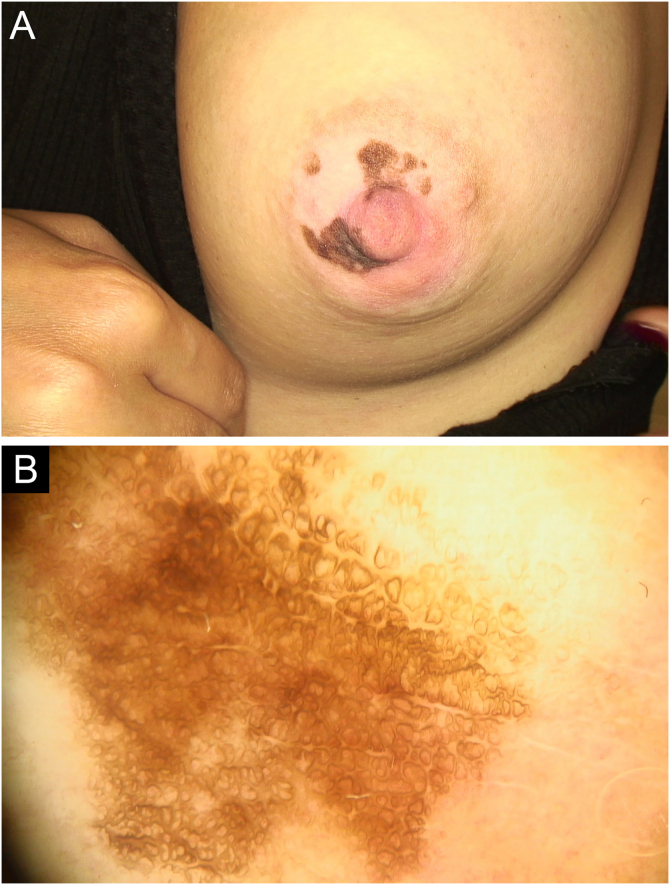
(A) Two irregularly bordered hyperpigmented patches on the nipple-areola complex in the first patient. (B) Dermoscopic image showing a pigmented background with circular structures forming a ring pattern in the first patient.

**Fig. 2:**
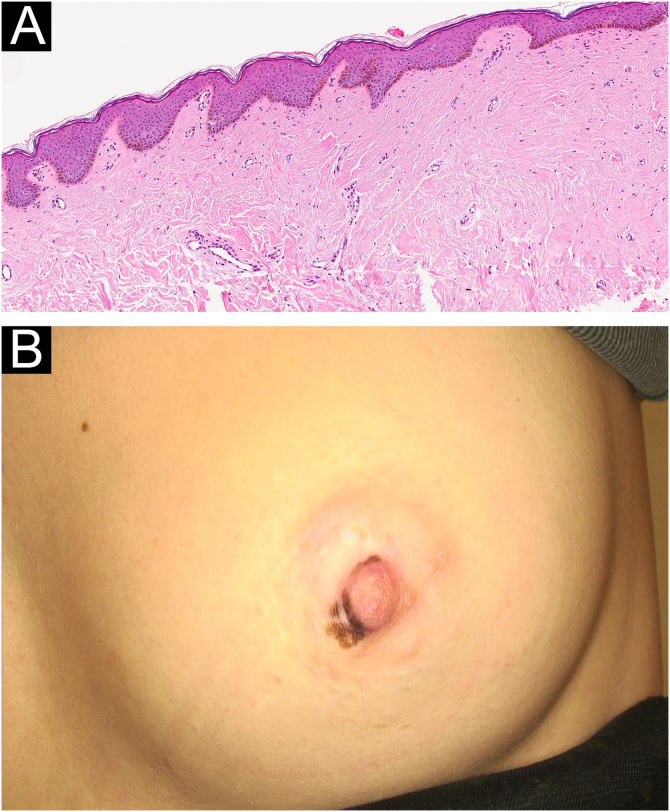
(A) Histopathological findings of the first patient: basal melanin hyperpigmentation, epidermal acanthosis, and elongation of rete ridges (Hematoxylin & eosin, ×10). (B) Partial regression of the lesions after four sessions of superficial cryotherapy in the first patient.

A 34-year-old female presented with irregular pigmentation on the right areola, which developed during the second trimester of pregnancy. She had a history of full-body tanning bed exposure one year prior, consisting of six sessions. Examination revealed a 3 cm hypopigmented patch with irregularly pigmented plaques (Fig. 3A). Dermoscopy demonstrated a pigmented background with circular structures and V-shaped linear pigmentation (fish-scale-like pattern) (Fig. 3B). Histopathology confirmed melanoacanthoma, showing epidermal acanthosis, rete ridge elongation, basal layer hyperpigmentation, superficial dermal edema, melanin incontinence, and perivascular lymphocytic infiltration (Fig. 4A). Over five years of follow-up, the lesion exhibited partial depigmentation without treatment, with no progression observed (Fig. 4B).

**Fig. 3:**
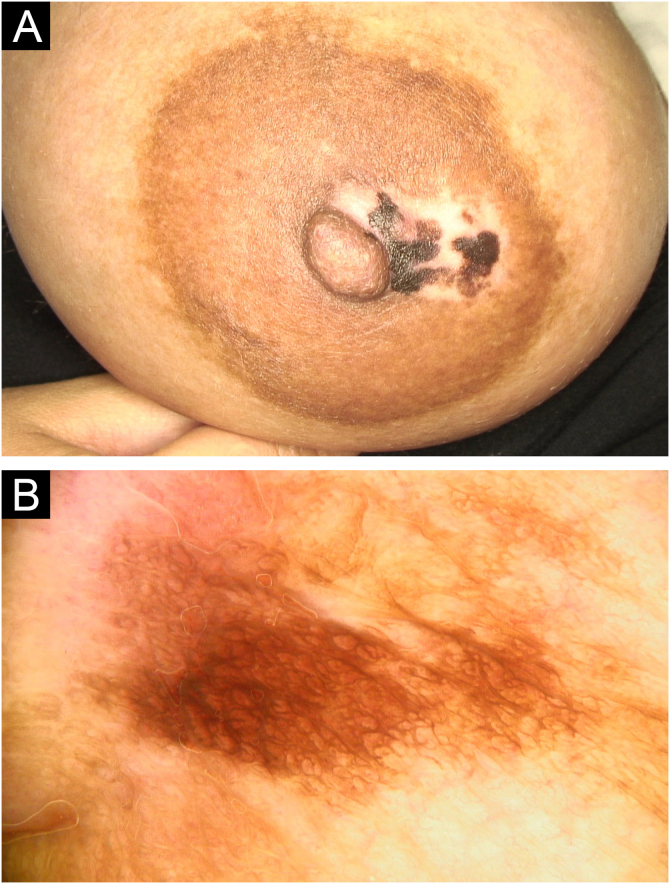
(A) A 3 cm hypopigmented patch interspersed with irregularly pigmented macules on the areola in the second patient. (B) Dermoscopic image showing circular structures forming a ring pattern and a fish scale-like pigmentation variant in the second patient.

**Fig. 4:**
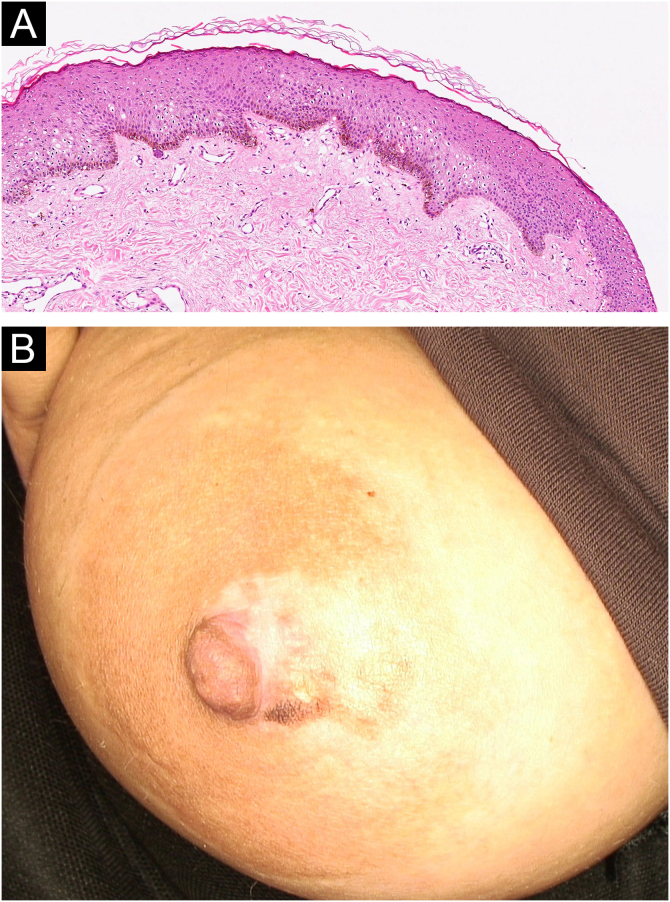
(A) Histopathological findings of the second patient: irregular acanthosis, elongation of rete ridges, increased basal melanin, dermal edema, melanin incontinence, and perivascular lymphocytic infiltration (Hematoxylin & eosin, ×10). (B) Partial regression of the lesion without treatment after five years in the second patient.

Melanoacanthoma is a heavily pigmented variant of seborrheic keratosis, commonly found on the head, neck, and trunk, with a predilection for older individuals.^1^ Its occurrence on the nipple-areola complex is extremely rare, with only two previously reported cases.^2^, ^3^ Proposed etiological factors include FGFR3 mutations, ultraviolet exposure, and hormonal influences such as pregnancy or estrogen therapy.^3^ Despite being a non-sun-exposed area, both of our patients had a history of tanning bed use, and one developed the lesion during pregnancy.

Differential diagnoses include nevoid hyperkeratosis, seborrheic keratosis, and melanoma.^2^, ^4^ Various patterns, including the starburst appearance, ridges, fissures, comedo-like openings, hairpin vessels, and sharp demarcation, have been described, along with melanoma-specific features such as a blue-white veil and atypical dots.^1^, ^2^, ^3^, ^4^, ^5^ In our cases, dermoscopy revealed circular structures, which had not been previously described in melanoacanthoma but have been observed in other benign lesions like labial melanotic macules.^6^

Histopathology remains essential for confirming melanoacanthoma, showing basaloid keratinocyte proliferation and scattered dendritic melanocytes. Given the aesthetic and functional considerations of the nipple-areola complex, biopsy should be carefully planned.^2^, ^3^ Most cases do not require treatment unless symptomatic; cryotherapy resulted in partial regression in one of our patients.^1^

These cases emphasize the importance of considering melanoacanthoma in the differential diagnosis of pigmented lesions in the nipple-areola region and highlight the need for dermoscopic and histopathologic evaluation for accurate diagnosis. The presence of circular structures expands its known features, and the history of UV exposure in both patients suggests a potential role in its pathogenesis.

## ORCID IDs

Aysun Sikar Akturk: 0000-0002-8137-2126; Nilgün Sayman: 0000-0003-3416-7987; Rebiay Kiran: 0000-0002-9515-1682; Cigdem Vural: 0000-0002-9405-9112

## Authors' contributions

Aysun Sikar Akturk: Approval of the final version of the manuscript; critical literature review; data collection, analysis and interpretation; effective participation in research orientation; ıntellectual participation in propaedeutic and/or therapeutic management of studied cases; manuscript critical review; preparation and writing of the manuscript; statistical analysis; study conception and planning.

Esin Diremsizoglu: Approval of the final version of the manuscript; critical literature review; data collection, analysis and interpretation; effective participation in research orientation; ıntellectual participation in propaedeutic and/or therapeutic management of studied cases; manuscript critical review; preparation and writing of the manuscript; statistical analysis; study conception and planning.

Nilgün Sayman: Approval of the final version of the manuscript; critical literature review; data collection, analysis and interpretation; effective participation in research orientation; ıntellectual participation in propaedeutic and/or therapeutic management of studied cases; manuscript critical review; preparation and writing of the manuscript; statistical analysis; study conception and planning.

Rebiay Kiran: Approval of the final version of the manuscript; critical literature review; data collection, analysis and interpretation; effective participation in research orientation; ıntellectual participation in propaedeutic and/or therapeutic management of studied cases; manuscript critical review; preparation and writing of the manuscript; statistical analysis; study conception and planning.

Cigdem Vural: Approval of the final version of the manuscript; critical literature review; data collection, analysis and interpretation; effective participation in research orientation; ıntellectual participation in propaedeutic and/or therapeutic management of studied cases; manuscript critical review; preparation and writing of the manuscript; statistical analysis; study conception and planning.

## Financial support

This research received no specific grant from any funding agency in the public, commercial, or not-for-profit sectors.

## Research data availability

Does not apply.

## Conflicts of interest

None declared.
